# Sphingosine 1 phosphate promotes hypertension specific memory T cell trafficking in response to repeated hypertensive challenges

**DOI:** 10.3389/fphys.2022.930487

**Published:** 2022-09-07

**Authors:** Maha M. Itani, Hala Jarrah, Dina Maaliki, Zeina Radwan, Rima Farhat, Hana A. Itani

**Affiliations:** ^1^ Department of Pharmacology and Toxicology, Faculty of Medicine, American University of Beirut, Beirut, Lebanon; ^2^ Department of Anatomy, Cell Biology and Physiology, Faculty of Medicine, American University of Beirut, Beirut, Lebanon; ^3^ Division of Clinical Pharmacology, Department of Medicine, Vanderbilt University Medical Center, Nashville, TN, United States

**Keywords:** inflammation, memory T cells, S1PR1, memory T-lymphocytes trafficking, kidney, hypertension

## Abstract

We have previously shown that effector memory (TEM) cells accumulate in the bone marrow (BM) and the kidney in response to l-NAME/high salt challenge. It is not well understood if measures to block the exodus of that effector memory cells prevent redistribution of these cells and protect from hypertension-induced renal damage. We hypothesized that that effector memory cells that accumulate in the bone marrow respond to repeated salt challenges and can be reactivated and circulate to the kidney. Thus, to determine if mobilization of bone marrow that effector memory cells and secondary lymphoid organs contribute to the hypertensive response to delayed salt challenges, we employed fingolimod (FTY720), an S1PR1 functional antagonist by downregulating S1PR, which inhibits the egress of that effector memory cells used effectively in the treatment of multiple sclerosis and cardiovascular diseases. We exposed wild-type mice to the l-NAME for 2 weeks, followed by a wash-out period, a high salt diet feeding for 4 weeks, a wash-out period, and then a second high salt challenge with or without fingolimod. A striking finding is that that effector memory cell egress was dramatically attenuated from the bone marrow of mice treated with fingolimod with an associated reduction of renal that effector memory cells. Mice receiving fingolimod were protected from hypertension. We found that wild-type mice that received fingolimod during the second high salt challenge had a marked decrease in the renal damage markers. CD3^+^ T cell infiltration was significantly attenuated in the fingolimod-treated mice. To further examine the redistribution of bone marrow that effector memory cells in response to repeated hypertensive stimuli, we harvested the bone marrow from CD45.2 mice following the repeated high salt protocol with or without fingolimod; that effector memory cells were sorted and adoptively transferred (AT) to CD45.1 naïve recipients. Adoptively transferred that effector memory cells from mice treated with fingolimod failed to home to the bone marrow and traffic to the kidney in response to a high salt diet. We conclude that memory T cell mobilization contributes to the predisposition to hypertension and end-organ damage for prolonged periods following an initial episode of hypertension. Blocking the exodus of reactivated that effector memory cells from the bone marrow protects the kidney from hypertension-induced end-organ damage.

## Introduction

Being one of the most seriously debilitating medical conditions, hypertension has been among the most studied topics. According to Imperial College London and the World Health Organization’s (WHO) first comprehensive global analysis of trends in hypertension, the number of hypertensive patients increased from 648 million in 1990 to 1.296 billion in 2019 (NCD Risk Factor Collaboration NCD-RisC, 2021). In the United States, nearly half of the adults (116 million) are hypertensive, with systolic or diastolic blood pressure equal to or greater than 130/80 mmHg, based on the newly released guidelines that redefined hypertension (Control CfD Prevention, 2019). High blood pressure continues to be the greatest single risk factor contributing to global disease burden and all-cause mortality. Statistically, hypertension is responsible for 19.2% of deaths in 2015 due to cardiovascular disease (CVD) comorbidities such as stroke, ischaemic heart disease, other vascular diseases, and renal disease (Forouzanfar et al., 2017).

Over the last decade, an extensive literature has developed to understand the role of the immune system, especially adaptive immunity, as a key contributor to the development of hypertension. In response to hypertensive stimuli, specific immune cells infiltrate the blood pressure-regulating organs like the kidney and the vasculature and contribute to end-organ damage (Drummond et al., 2019). Several researchers have discovered that animals missing adaptive immune cells exhibit attenuated hypertension in response to stimuli including angiotensin II (ANG II), excessive salt, and norepinephrine, while adoptive transfer of T cells restores the blood pressure response to these stimuli (Drummond et al., 2019). This has been highlighted by several studies, with a focus on the cytotoxic T lymphocytes (CD8^+^) and T helper cells (CD4^+^) (Youn et al., 2013; Itani et al., 2016a; Zubcevic et al., 2017). CD8^+^ cells influence blood pressure by enhancing renal sodium and water reabsorption and secreting cytokine Interferon *γ* (IFNγ) upon kidney infiltration (Saleh et al., 2015; Liu et al., 2017) and by promoting endothelial dysfunction upon vasculature infiltration (Trott et al., 2014). Besides, we previously reported infiltration of human leukocytes, including CD4^+^ cells, into the thoracic lymph nodes, thoracic aorta, and kidneys in hypertensive humanized mice (Itani et al., 2016a). A potential mechanism by which CD4^+^ cells contribute to hypertension is that these cells secrete inflammatory cytokines IFNγ and Interleukin-17 (IL-17) (Bestetti et al., 2019), as mice deficient in these cytokines failed to secrete these cytokines and develop hypertension in response to ANG II infusion (Madhur et al., 2010; Kamat et al., 2015; Saleh et al., 2016).

A special hallmark of the adaptive immune system is the immunological memory, in which a clone of antigen-specific lymphocytes persists for a lifetime, protecting against repeated antigenic exposure. When T lymphocytes encounter an antigen, they initiate an immune response. After its elimination, effector T cells die except for a few remaining cells, which become memory T cells mounting immediate recall responses upon re-exposure to the same antigen. Many existing studies in the broader literature have examined subpopulations of memory T cells: effector memory T cells (CD44^hi^ CD62L^lo^) and central memory T cells (CD44^hi^ CD62L^hi^). Effector memory T cells (T_EM_) patrol between blood and peripheral tissues and have immediate effector action.

On the contrary, the central memory T cells (T_CM_) are found in secondary lymphoid organs, have little or no effector function, but differentiate into effector cells in response to antigenic stimulation. These cells have been extensively studied in humans’ pathophysiological genesis of hypertension. To illustrate, our previous data showed that in hypertensive mice, memory T cells are important sources of IFNγ and IL-17A, and these cells appear to trigger elevated blood pressure in response to repeated hypertensive stimuli (Itani et al., 2016b). Similarly, another study reported an increase in circulating memory T cells in the blood of hypertensive mice (Itani et al., 2016a).

An extensively studied molecule in the pathology of hypertension and its associated cardiovascular events is sphingosine 1-phosphate (S1P). Although there is a need to validate the S1P-blood pressure in humans, Jujic et al. suggested the therapeutic potential of S1P as a diagnostic biomarker in a translational approach (Jujic et al., 2021). A closer look at the literature on T lymphocyte recirculation in hypertensive models reveals the role of bioactive lipid molecules S1P and S1PR1 (Don-Doncow et al., 2019). Drouillard et al. claimed that T cells’ migratory behavior depends on the interaction between S1P and sphingosine 1-phosphate receptors 1 and 2 (S1PR1-2) with higher affinity towards S1PR1 (Drouillard et al., 2018). Another study showed the importance of S1PR1 in migrating T cells from the secondary lymphoid organs. Loss of expression on CD4 and CD8 splenic T cells from THI- and DOP- treated mice blocked the cell’s migration in response to S1P (Schwab et al., 2005). In mammals, S1P molecules are compartmentalized with a high concentration in blood and lymph while undetectable in lymphoid organs. This S1P gradient and S1PR1 expression levels are particularly important for the egress of T cells into blood by S1PR1-dependent sensing of S1P gradients, causing the lymphocyte to egress from the secondary lymphoid organs into the systemic circulation (Matloubian et al., 2004; Schwab et al., 2005). Once in circulation, the interaction between S1P molecules and S1PR1 enhances ligand-receptor internalization and breaks the S1P and S1PR1 gradient, causing the T cells to bypass the S1P-dependent attraction to blood (Sasset and Di Lorenzo, 2022). S1P has a physiological function in immune modulation that has been unraveled by elucidating the mechanism of Fingolimod (FTY720), effective treatment for multiple sclerosis and cardiovascular diseases (Lucaciu et al., 2020a). Pharmacologically, FTY720 is a safe and effective second-line therapeutic agent to treat multiple sclerosis, a chronic auto-immune disease, by diminishing annualized relapse rate, risk of disability progression and inflammatory activity of relapsing MS (Fazekas et al., 2013; Behjati et al., 2014). In addition to its effect on inflammatory responses and immune cells, FTY720 has protective effect on the cardiovascular system, for examples, it improves the cardiac function and lowers blood pressure (Camm et al., 2014; Ahmed et al., 2019). Therefore, here in, FTY720 was taken as a be a key pharmacological agent to block memory T cells in hypertensive mice. When taken on a long-term basis, Fingolimod causes S1PR1 to be internalized and degraded, making it a “functional antagonist.” Fingolimod acts as a prodrug, which is intracellularly phosphorylated, binding to all S1PRs except for S1PR2 with a preference for S1PR1 (Cartier and Hla, 2019; Lucaciu et al., 2020a). As a result, fingolimod depletes lymphocytes in the peripheral circulation and sequesters them in secondary lymphoid organs. Interestingly, Dominguez-Villar et al. reported an important immunomodulatory function of fingolimod besides altering T cell trafficking. The data imply that fingolimod changes the T cells’ phenotype into an exhausted-like phenotype, characterized by reduced IL-17 and IFN-γ expression (Dominguez-Villar et al., 2019).

Interestingly, when fingolimod was approved as an effective treatment for multiple sclerosis, diverse cardiovascular events started to be reported suggesting a link between the role of S1P signaling and BP regulation. A novel study supported this point and reported S1P molecules as systemic blood pressure regulators via S1PR1–S1P–eNOS autocrine signaling (Cantalupo and Di Lorenzo, 2016). Besides, intravenous treatment of FTY720 caused a transitory drop in heart rate (HR) and blood pressure (BP) in anesthetized rats and humans during phase 3 clinical trials (Intapad, 2019). Having a role in immunomodulation and hypertension regulation, there is a need to study the mechanisms by which fingolimod regulates blood pressure by controlling immune cell trafficking.

To our knowledge, the best-known fingolimod mode of action is the blockade of T_CM_ cells in the lymph node without impairing the circulation of T_EM_ cells. This strategy prevents the production of new self-reactive lymphocyte clones that sustain the disease while maintaining pathogen defense (Balandina et al., 2005). For this study, it was of interest to identify the molecular mechanisms by which memory T cells contribute to inflammation and hypertension in response to repeated high salt stimuli. Herein, we hypothesized that S1PR1 plays an essential role in the egress of T_EM_ cells from the bone marrow to the kidney and that pharmacological blockade of S1PR1 by fingolimod prevents renal damage and hypertension onset in response to repeated high salt stimuli. Therefore, we aimed to determine the role of T cell S1PR1 in hypertension in response to repeated hypertensive challenges. We successfully analyzed the role of S1PR1 in hypertension-induced end-organ damage by detecting secreted pro-inflammatory cytokines secreting memory T cells infiltrating the kidney. Moreover, we highlight the role of S1PR1 in the reactivation and redistribution of T_EM_ cells in response to repeated antigenic exposure through the employment of the adoptive transfer protocol.

## Methods

### Animals and study design

Animals were housed at the American University of Beirut (AUB) Animal Care Facility (ACF). The mice were housed at 25°C on a 12 h light/dark cycle throughout the study. Mice had access to water *ad libitum* and maintained a regular Teckled diet. All procedures were approved by the Animal Care Facility Animal Ethics Committee and conducted according to the IACUC Guidelines for the Ethical Use of Animals in research, AUB (16 July 2018). All experimental procedures and analysis were done.

CD45.2 wild-type (WT) male C57BL/6 mice were purchased from Jackson Laboratories and studied at 12 weeks. For this study, a minimum number of 6 WT mice were randomly assigned and used per group. WT mice received the L-NAME + HS1+HS2 protocol with and without fingolimod (fingolimod). Briefly, all mice at 12 weeks of age received l-NAME N(G)-Nitro-l-arginine methyl ester (l-NAME) 0.5 mg/ml (Cayman) in the drinking water for 2 weeks high-salt protocol (l-NAME/HS1) as previously described (Machnik et al., 2009). Then mice were shifted to regular drinking water for 2 weeks, referred to as the washout period (WO). Following that, the mice were fed high salt diet (4% NaCl, Harlan, United States) for 4 weeks and then shifted to the WO period. Finally, the mice were fed for the second time high salt diet (4% NaCl, Harlan, United States) over 4 weeks, during which 6 of them received the fingolimod treatment every other day. Treated groups of the mice received fingolimod (1 mg/kg) IP every other day during this time compared to six control mice infused with saline.

The mortality rate was 0, and none of the mice died throughout the protocol until the day of the sacrifice.

### Blood pressure measurements

Invasive method (Radiotelemetry): Blood pressure was measured invasively using radio-telemetry for WT mice. Mice were given 2 weeks to recover after telemetry implantation before beginning the LNAME/high salt protocol with and without fingolimod treatment. Previous data show the BP throughout the LN-NAME + HS1 protocol (Itani et al., 2016b). Thus, and to preserve the battery, telemetric blood pressure measurements were recorded at the end of the HS2 throughout the treatment of fingolimod (Stellar Telemetry, TSE Systems, United States) as described previously (Muntner et al., 2018).

Non-invasive method (Tail-cuff method): In addition to that, a non-invasive method, through the BP-2000 Blood Pressure Analysis System™, was utilized. Initially, we gave the mice about a week to adapt to the machine before taking baseline readings. After that, we measured the blood pressure of WT mice and CD45.1 mice thrice per week after a successful adoptive transfer.

### Renal damage assessment

Kidney damage was evaluated in WT mice towards the end of the LNAME + HS1 + HS2 ± fingolimod protocol. After the sacrifice, one of the two isolated kidneys was dissected into two equal parts. The first half was used for immunohistochemical analysis of CD3^+^ T-cells expression in mice kidney tissue. While the other half was used to quantify the levels of NGAL, which is a marker for renal damage.

*Immunohistochemistry and Imaging:* Harvested kidneys were fixed with formaldehyde and cross-sectionally sectioned into 5 μm slides for staining. 10% formalin-fixed, paraffin-embedded tissue sections of kidneys were sectioned at five- um thickness. For anti-CD3 staining: Slides were deparaffinized. Heat-induced antigen retrieval was performed using the Epitope Retrieval two solution for 10 min. Slides were incubated with a monoclonal rabbit anti-mouse CD3 antibody (anti-CD3 (ab16669, Abcam, Cambridge, MA) at a 1:250 dilution for 60 min. The Bond Polymer Refine Detection system was used for visualization. Slides were then dehydrated, cleared, and coverslipped. Quantification of CD3 positive cells in the renal cortex was conducted by Image J software.

*Quantitative Real-time PCR for NGAL Expression Analysis:* Total RNA was extracted from half a kidney harvested from each mouse in the two groups (±fingolimod) using Trizol (15596018). The extracted RNA was quantified and reverse transcribed into cDNA using the High Capacity cDNA Reverse Transcription kit (Applied Biosystems, Foster City, CA, United States) following the manufacturer’s instructions. To detect NGAL mRNA expression, RT-qPCR (BioRad CFX384 Real-Time System, C1000 thermal cycler, Hercules, CA, United States) was carried out for each sample in duplicates, using 2x SensiFAST SYBR No-ROX Mix (Bioscience, Cincinnati, OH, United States), following the manufacturer’s instructions. The cycling conditions were 2 min at 95°C, then 40 cycles of 5 s at 95°C (denaturing step) and 60 s at 60.3°C (annealing and extension steps). GAPDH was used as an endogenous control. Therefore, the relative expression of NGAL was normalized to the chosen endogenous control and was compared to that group of mice that did not receive fingolimod treatment. ∆∆Ct method was used to calculate the relative fold change of expression. The primers were obtained from Macrogen (Seoul, Republic of Korea).

### Flow cytometry analysis

Single-cell suspensions of the bone marrow and kidney from WT (L-NAME + HS1 + HS2 ± fingolimod) and CD45.1 mice were prepared as previously described (Saleh et al., 2015). Kidneys were mechanically dissociated using a gentleMACS C tube (Miltenyi Biotech, Bergisch Gladbach, Germany) in combination with a gentleMACS dissociator system (Miltenyi Biotech, Bergisch Gladbach, Germany), followed by incubation at 37°C for 20 min with collagenase D (Roche Diagnostics, Mannheim, Germany) (2 mg/ml) and DNAse I (Roche Diagnostics, Mannheim, Germany) (100 μg/ml) in RPMI 1640 medium with 5% FBS while being gently rotated in a hybridization oven. Kidney homogenates were filtered through 70 μm cell strainers. Percoll™ PLUS (GE Healthcare, Upsala, Sweden) subjected the resultant cell suspensions to gradient centrifugation. Cells were isolated from the Percoll interface and washed in cold PBS. As for the bone marrow tissue, the cells were isolated from the Tibias and femurs of mice into RPMI 1640 media by centrifuging the bones at 17,7750 x G for 5 min and then filtered through 40 µm strainers. Red blood cells were osmotically ruptured by RBC lysis buffer (Invitrogen, Van Allen Way, Carlsbad, CA, United States). Isolated cells were washed in cold PBS. After that, one million cells were counted using trypan blue and assigned for flow cytometry analysis, while the remaining cells were used to isolate T cells, as mentioned previously. 10 million cells were counted using trypan blue for the adoptive transfer.

*Extracellular staining:* Single-cell suspensions were washed and stained LIVE/DEAD® Fixable Violet dead cell stain (Invitrogen, Eugene, OR, United States). The following antibodies and fluorophores were used: Brilliant Violet 510 (BV510)-conjugated anti-CD45 antibody, peridinin chlorophyll protein-cyanin-5.5 (PerCP-Cy5.5)-conjugated anti-CD3 antibody, allophycocyanin-cyanin-7 (APC-Cy7)-conjugated anti-CD4 antibody, phycoerythrin-cyanin-7(PE-Cy7)-conjugated anti-CD8a antibody, APC-conjugated anti-CD44 antibody, and PE-conjugated anti-CD62L antibody, and FITC anti-F4/80 (BioLegend, San Diego, CA, United States). Before analysis, we add 50 µL of 1,2,3 count eBeadsTM counting beads (Invitrogen, Van Allen Way, Carlsbad, CA, United States) to each sample, except for the cells isolated from the bone marrow. Samples were run on a BD FACS Aria SORP cell sorter and analyzed using FlowJo software V10 (Tree Star inc.). Gates were identified using fluorescence minus one (FMO). Results were normalized using the bead count and expressed as a number of cells per kidney. All lymphocyte subpopulations (CD4^+^, CD8^+^) were quantified within the CD45^+^CD3^+^ gate. Harvested T cells, including naïve cells, T_CM_, and T_EM,_ were quantified. The role of S1PR1 in T_EM_ cells trafficking from the bone marrow to the kidney in response to a hypertensive stimulus was examined by performing the adoptive transfer. After being fed the high salt diet for 3 weeks, T cells adoptively transferred from CD45.2 mice, expressing either free S1PR1 or pharmacologically blocked S1PR1 by FTY620, were examined in the kidney and bone marrow of CD45.1 recipient mice. *Intracellular staining:* Kidney cell suspensions were prepared as previously described. Cells were washed and stained first with LIVE/DEAD® Fixable Violet dead cell stain (Invitrogen, Eugene, OR, United States). Intracellular staining was then performed with the Fix and Perm Cell Permeabilization kit (life technologies) following the manufacturer’s instructions and using FITC conjugated anti–IFN-γ antibody (eBioscience, clone XMG1.2) and anti IL17. The following surface antibodies were then added for 30 min: Brilliant Violet 510 (BV510)-conjugated anti-CD45 antibody, peridinin chlorophyll protein-cyanin-5.5 (PerCP-Cy5.5)-conjugated anti-CD3 antibody, allophycocyanin-cyanin-7 (APC-Cy7)-conjugated anti-CD4 antibody, phycoerythrin-cyanin-7(PE-Cy7)-conjugated anti-CD8 antibody, APC-conjugated anti-CD44 antibody (Biolegend San Diego, CA, United States).

### T cell isolation and adoptive transfer

In adoptive transfer experiments, WT mice (CD45.2 donors, Jackson Laboratories) were subjected to the L-NAME + HS1+HS2±fingolimod protocol described above. Towards the end of the protocol, T cells were isolated from the bone marrow using Pan T Cell Isolation Kit II mouse (Miltenyi Biotech, Bergisch Gladbach, Germany) and negative magnetic sorting through MidiMACS™ Separator from Miltenyi Biotech. Sterile and highly enriched T cells were obtained. 10 million cells were suspended in 1.5 ml saline solution and transferred to the CD45.1 recipient mice through tail vein injection. The mice were left to recover for 1 week, in which they were fed a normal diet, followed by 3 weeks of a high salt diet (4%NaCl, Harlan, United States). At the end of all experiments, the mice were sacrificed by CO_2_ inhalation. Blood pressure was measured non-invasively using the same protocol as mentioned above. Similarly, single-cell suspension of bone marrow and kidney from CD45.1 recipient mice were stained for CD4^+^ and CD8^+^, T_EM,_ and T_CM_ cells, as mentioned in the flow cytometry analysis (extracellular staining).

Statistics: All statistical analysis was completed using GraphPad Prism version 8.0.0 (California, United States). Data are presented as mean ± standard error of the mean (SEM). For repeated measures, 2-way ANOVA was used to analyze blood pressure measurements. Two-way ANOVA was used to compare experiments involving a 2 × 2 design. Newman-Keuls or Holm-Sidak posthoc tests were employed for individual comparisons within this 2 × 2 design. Mann Whitney comparison followed by a Bonferroni correction was used when unequal variances between groups. *p* values less than or equal to 0.05 were considered significant.

## Results

### Fingolimod reduces hypertension

**FIGURE 1:**
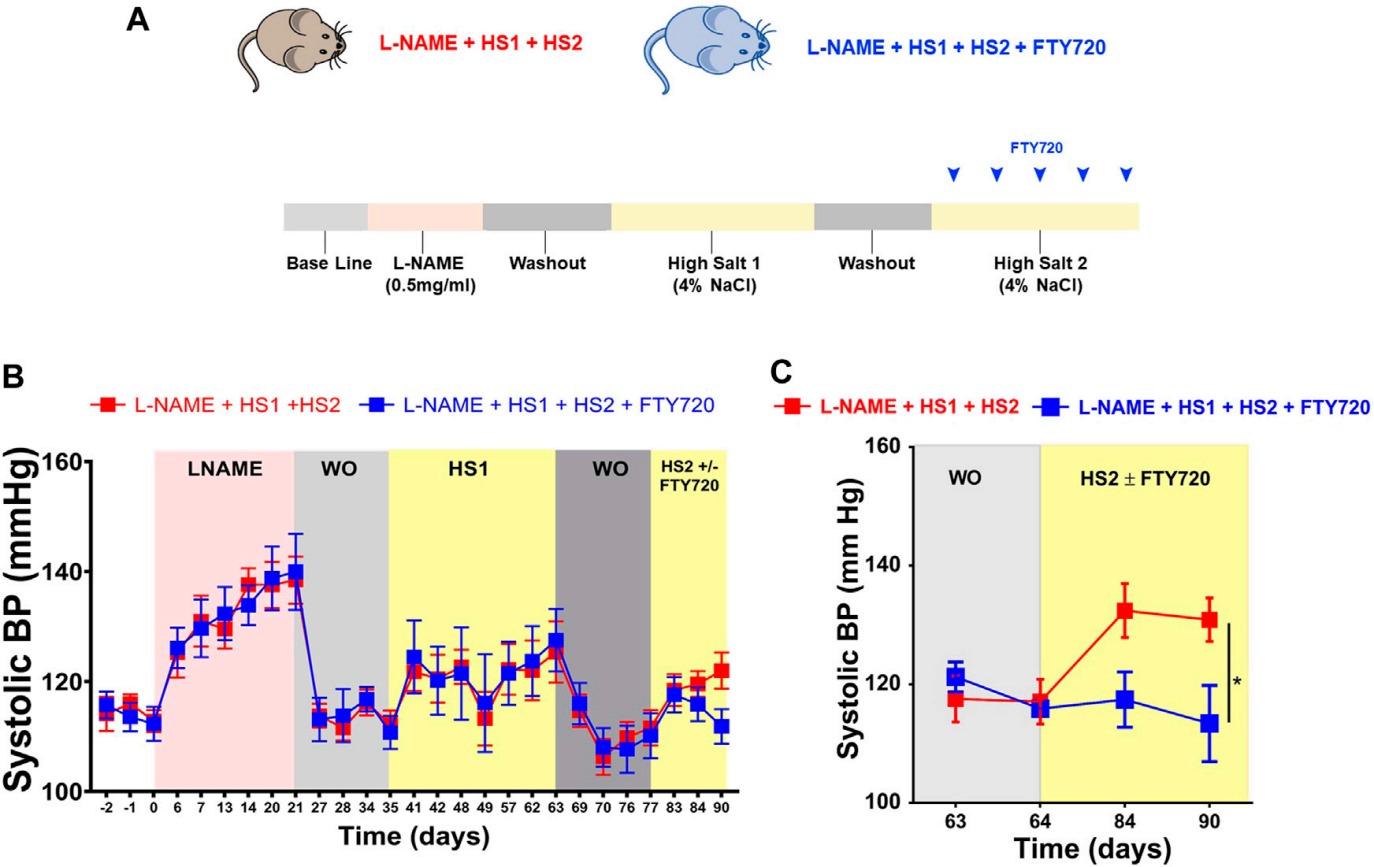
Fingolimod protects the mice from developing hypertension after exposure to the same hypertensive stimulus (high salt diet). **(A)** Experimental paradigm: summary of the protocol followed describing the diet fed to the mice at each phase during the experiment. **(B)** Systolic blood pressure of conscious mice that received L-NAME + HS1+HS2±fingolimod was measured using the non-invasive tail-cuff method. **(C)** Systolic blood pressure of conscious mice that received L-NAME + HS1+HS2±fingolimod was measured using the invasive radiotelemetry method. N = 6–10 mice/group. Results are presented as mean ± SEM. *p*-value calculated by 2-way Anova test. *p < 0.05.

C57BL/6 mice were divided into two groups and placed on L-NAME + HS1 + HS2. One group of mice received fingolimod (FTY720,1 mg/kg) IP every other day during the second HS2 period (l-NAME/HS1HS2 ± fingolimod). This model permits repeated hypertensive challenges without surgical interventions and recapitulates salt-sensitive hypertension common in humans (Figure 1A). To determine the role of T cell S1PR1 in hypertension in response to repeated hypertensive challenges, tail-cuff BP measurement showed that the systolic blood pressure increases gradually upon the administration of l-NAME and returns to baseline when l-NAME is discontinued. The BP increased again with high salt (HS1) and returned to the baseline at the second WO period; remarkably, the group receiving the high salt diet (HS2) alone showed an increase in BP, but the group that received fingolimod upon the second exposure to high salt (HS2) was protected from developing hypertension (Figure 1B). Similarly, telemetric blood pressure recordings on day 90, showed a significant increase in the blood pressure (*p*-value 0.04) of the mice that did not receive fingolimod in comparison to those that received fingolimod injections every other during the HS2 phase (SBP = 130.9130.9 mmHg vs. 113.5 mmHg, respectively). Therefore, the mice receiving fingolimod were protected from developing a hypertensive response (Figure 1C).

### Fingolimod reduces renal inflammation and prevents renal damage

**FIGURE 2:**
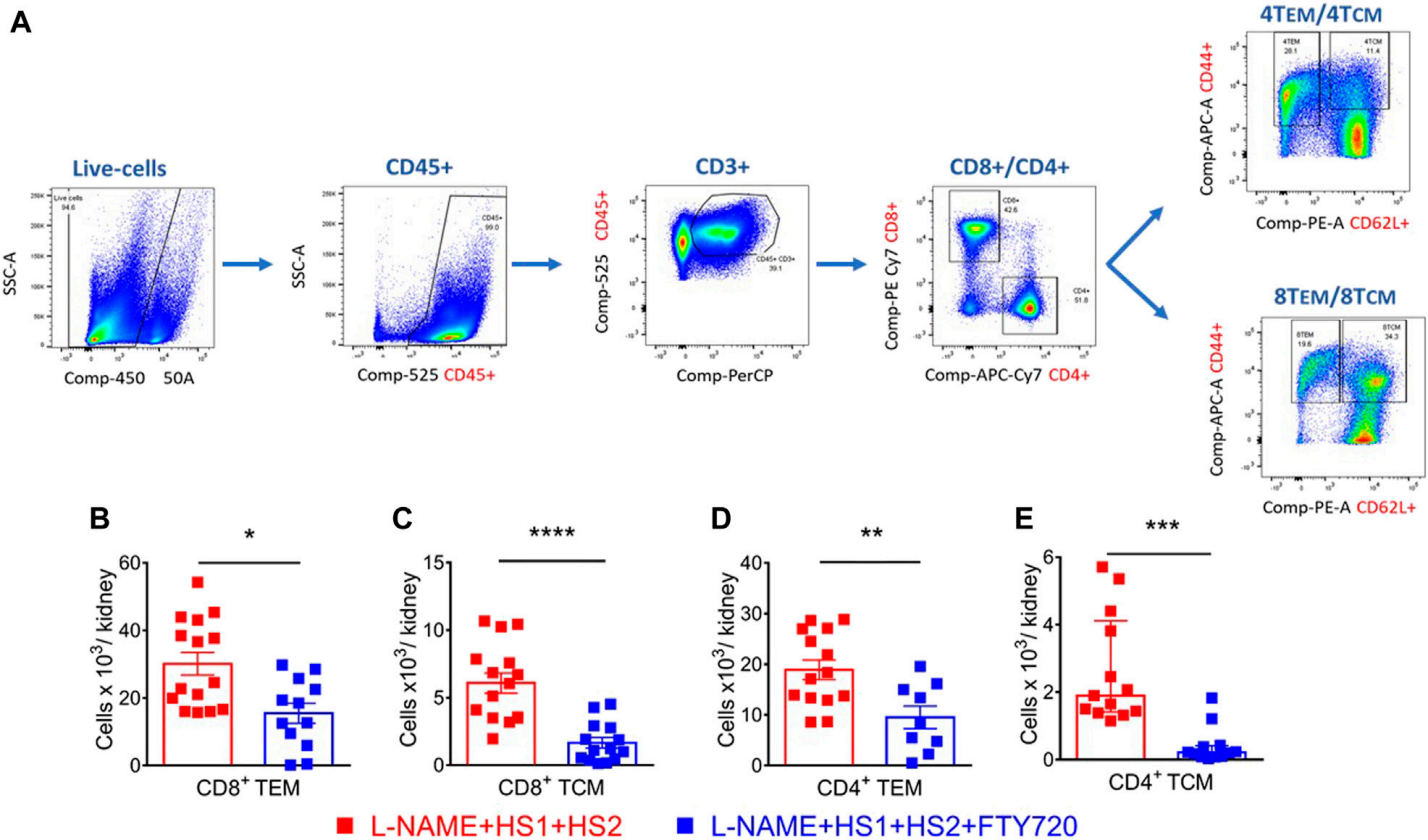
S1PR1 pharmacological blockade, via fingolimod, inhibited renal inflammation and protected from renal injury in response to a high salt diet. **(A)** Representative flow cytometric gating strategy of kidney single-cell suspensions showing the effect of l-NAME followed by high salt exposure on total leukocytes (CD45^+^ expression), total T lymphocytes (CD3^+^), total CD8^+^ and CD4^+^ T cells, CD8^+^ and CD4^+^ CD44hi/CD62Llo (TEM cells) and CD44hi/CD62Lhi (TCM cells). SSC-A indicates a side scatter area. **(B–E)** Summary of flow cytometric quantification of absolute numbers of CD8^+^ and CD4^+^ TEM and TCM cells infiltrating the kidney in WT mice that received L-NAME + HS1+HS2±fingolimod. N = 9–16 mice/group. Normally distributed data are expressed as mean ± SEM (panels B, C and D); not normally distributed data are expressed as median ± IQR (panel E) (Supplementary table 1). *p*-values calculated by unpaired *t*-test were shown. **p < 0.01, ***p < 0.001, ****p < 0.0001.

Studies showed that CD4^+^ and CD8^+^ T_EM_ cells increase in the kidney following l-NAME/high salt exposure and are responsible for producing injurious cytokines, including IL-17 and INFγ (Itani et al., 2016b). Here, we hypothesized that S1PR1 plays an essential role in the egress of T_EM_ cells from the bone marrow to the kidney and that S1PR1 pharmacological blockade via fingolimod prevents renal damage. In our gating strategy, we drill down from live singlets and stain for CD45^+^, CD3^+^, CD8^+,^ and CD4^+^ T cells, particularly for T_EM_ cells characterized by CD44^hi^ CD62L^Lo^ surface markers and T_CM_ cells which are characterized by CD44^hi^ Cd62L^hi^ surface markers (Figure 2A). Results of flow cytometry analysis showed that mice fed a high salt diet had a significantly higher level of CD8^+^ and CD4^+^ T_EM_ and T_CM_ cells infiltration to the kidney, an effect that is completely abrogated in the presence with fingolimod (*p*-values: CD8^+^ T_TEM_ 0.004; CD8^+^ T_TCM_ <0.0001; CD4^+^ T_TEM_ 0.0056; CD4^+^ T_TCM_ 0.0002) (Figures 2B–E). Flow cytometry results assure that S1PR1 pharmacological blockade inhibits the infiltration of T_EM_ cells to the kidney and thus protects from renal inflammation.

**FIGURE 3:**
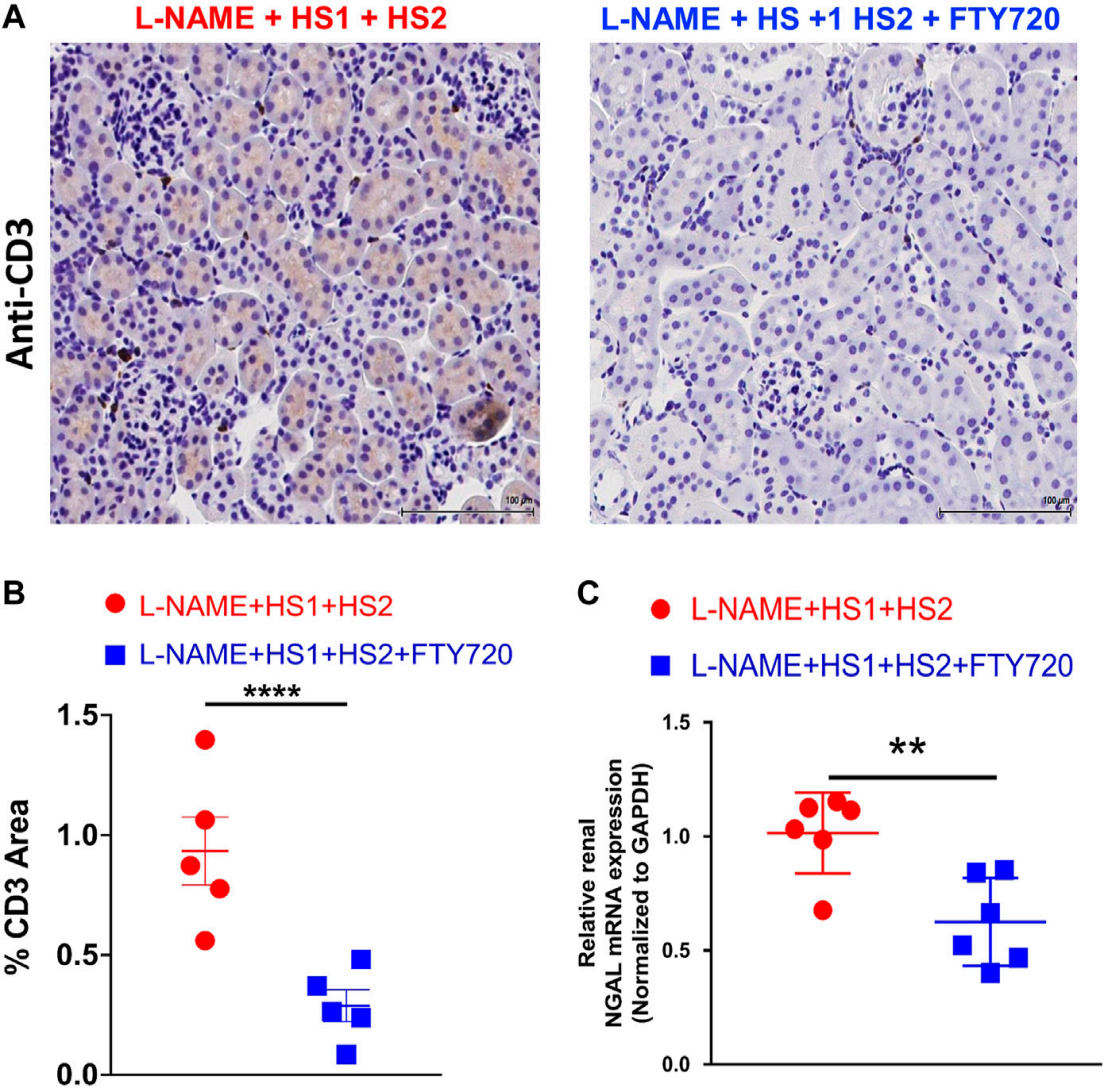
Fingolimod protects renal tissue from injury in mice following L-NAME + HS1+HS2±fingolimod protocol. **(A)** Immunohistochemical analysis of CD3^+^ T-cells expression in mice kidney tissue after receiving L-NAME + HS1+HS2±fingolimod. **(B)** Dot plot showing the percentage of CD3^+^ area in the renal tissue of the mice following L-NAME + HS1+HS2±fingolimod protocol. N = 5 mice/group. **(C)** Fold change of expression of NGAL relative to GAPDH in the kidney of mice that received L-NAME + HS1+HS2±fingolimod. N = 6 mice/group. Data are expressed as mean ± SEM. *p*-values calculated by unpaired *t*-test were shown. **p < 0.001, ***p < 0.0001.

Furthermore, kidney slides were stained with anti-CD3 antibody, and renal NGAL expression was detected to assess renal damage. Immunostaining with anti-CD3 revealed a striking renal infiltration of T cells in response to the LNAME + HS1+HS2 protocol, which was completely prevented by fingolimod (*p*-value < 0.0001), as shown in the representative microscopic images (Figure 3A) and related histograms (Figure 3B). Another criterion was adopted to assess kidney dysfunction: detecting the level of renal NGAL expression. Increased levels of NGAL have been linked to measurable damage in the loop of Henle and distal convoluted tubule, suggesting that it may have a predictive ability for kidney malfunction (De Silva et al., 2016). Thus, to further verify the protective role of fingolimod against renal damage in response to repeated salt exposure, NGAL expression levels were measured in both studied groups (L-NAME + HS1 + HS2 ± fingolimod). This analysis showed significant downregulation (*p*-value 0.0087) in the relative expression of NGAL in kidney organs isolated from mice that received fingolimod injections during the HS2 phase compared with mice that did not receive fingolimod injections (Figure 3C).

### Fingolimod controls memory T cells trafficking

**FIGURE 4:**
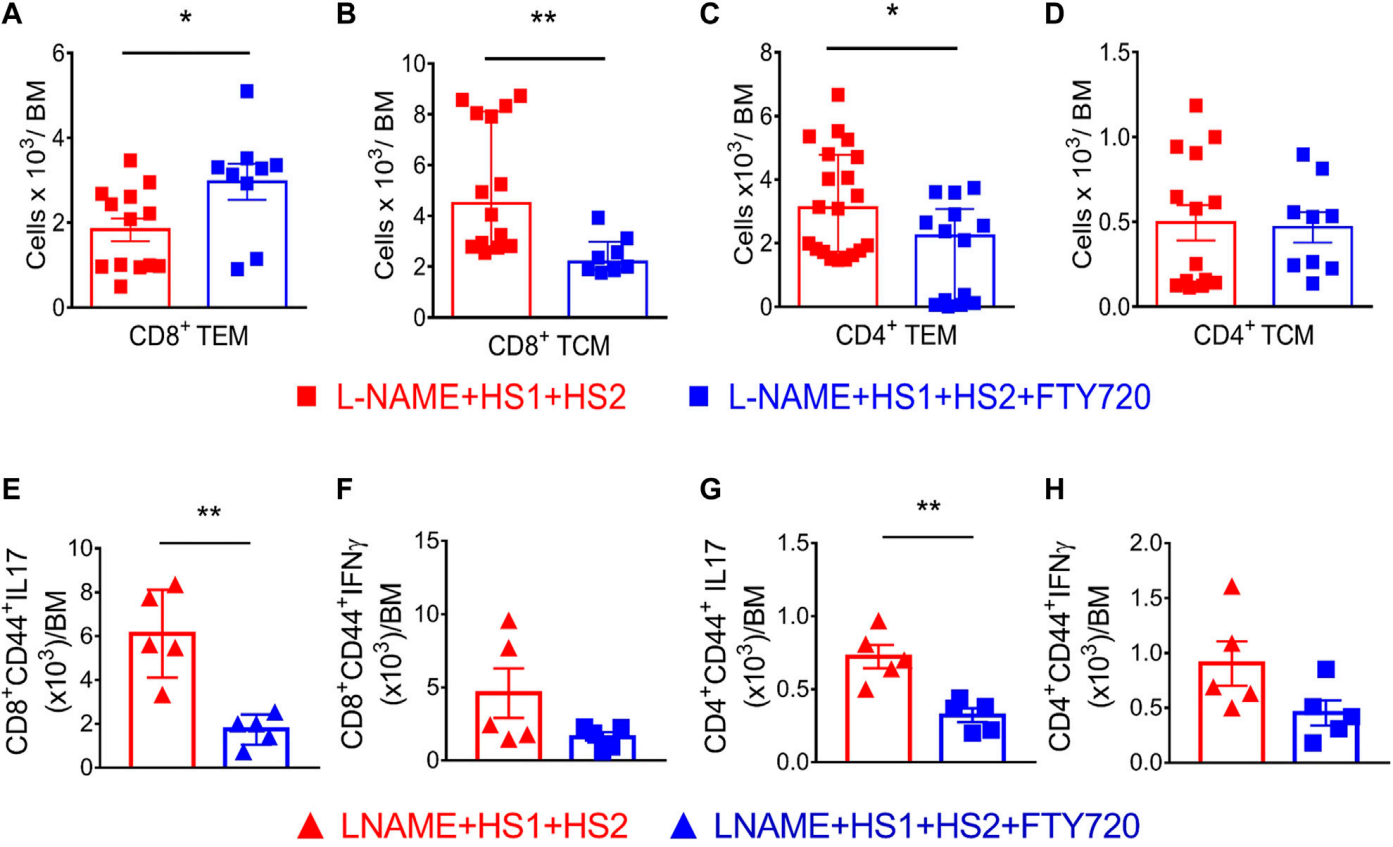
Fingolimod locks CD8^+^ and CD4^+^ TEM and TCM cells in the bone marrow and attenuates their secretion of inflammatory cytokines. **(A–D)** Flow cytometric quantification of total CD8^+^ (A&B) and CD4^+^ (C&D) TEM and TCMsinglengel-cell suspensions of the bone marrow from WT mice following the L-NAME + HS1+HS2±fingolimod protocol. N = 8–20 mice/group. Normally distributed data are expressed as mean ± SEM (panel A&D); not normally distributed data are expressed as median ± IQR (panel B&C) (Supplementary Table S2). **(E–H)** Intracellular staining showing the levels of secreted IL-17 and IFN-γ by CD4^+^ and CD8^+^ effector memory cells isolated from mice that received L-NAME + HS1+HS2±fingolimod. N = 5 mice/group. All data are normally distributed expressed as mean ± SEM (Supplementary Table S3). *p*-values calculated by unpaired *t*-test were shown. *p < 0.05, **p < 0.01.

Recent studies showed that some T_EM_ cells escape apoptosis following an initial hypertensive stimulus and remain in a quiescent state in the bone marrow. In addition, Itani et al. demonstrated a 1.8-fold increase in CD4^+^ T_EM_ cells and a 3-fold increase in CD8^+^ T_EM_ cells in the bone marrow following the l-NAME/high salt exposure (Itani et al., 2016b). Flow cytometry was performed to determine if effector memory cells are reactivated in response to a moderate hypertensive stimulus like salt. Fingolimod was able to block the exodus of these cells from BM following high salt activation single-cell suspensions from bone marrow from four groups of mice. One group received a normal diet, the other was exposed to l-NAME/high salt protocol, while the remaining two groups received the L-NAME + HS1 + HS2 ± fingolimod as previously explained. Interestingly, CD8^+^ CD44^hi^ CD62L^lo^ T_EM_ cells increase by 2 × 10^3^ folds in response to L-NAME + HS1 + HS2. This increase is even more significant in the presence of fingolimod compared to the group that did not receive fingolimod (*p*-value 0.0275) and reaches around 3 × 10^3^ folds (Figure 4A). On the other hand, a significant decrease in CD8^+^ CD44^hi^ CD62L^hi^ T_CM_ was reported in the group that received fingolimod (*p*-value 0.0074) (Figure 4B). CD4^+^ CD44^hi^ CD62L^lo^ T_EM_ cells increase significantly with L-NAME + HS1+HS2 and decrease with fingolimod (*p*-value 0.0102) (Figure 4C). No significant change was shown in CD4^+^ CD44^hi^ CD62L^hi^ T_CM_ following L-NAME + HS1+HS2 in the presence or absence of fingolimod (*p*-value 0.8501) (Figure 4D). These results assure the role of fingolimod in locking CD8^+^ T_EM_ cells but no other cells in the bone marrow. This indicates that blocking the S1PR1 receptor by fingolimod traps CD8^+^ effector memory cells in the BM and inhibits the recirculation into peripheral organs.

It has been demonstrated that effector memory cells are a major source of inflammatory cytokines, specially IFN-γ and IL-17A, in the kidney following l-NAME/HS stimulation (Itani et al., 2016b). Hence, we sought to determine if T_EM_ cells within the BM are responsible for producing injurious cytokines and if the S1PR1 blockade by fingolimod prevents the release of these inflammatory cytokines. Results of intracellular staining showed a significant decrease in the secretion of IL-17 by CD4^+^ and CD8^+^ effector memory cells isolated from mice injected with fingolimod compared to those not injected with fingolimod (*p*-value 0.0024 and 0.0017, respectively) (Figures 4E,G). As for IFN-γ, no significant difference was reported between the two groups (*p*-value 0.0881 and 0.1189, respectively) (Figures 4F,H). Thus, intracellular staining indicated that IL-17A production, not IFN-γ, was significantly abrogated in response to fingolimod following the L-NAME + HS1 + HS2 protocol.

### Fingolimod attenuates salt sensitivity

**FIGURE 5:**
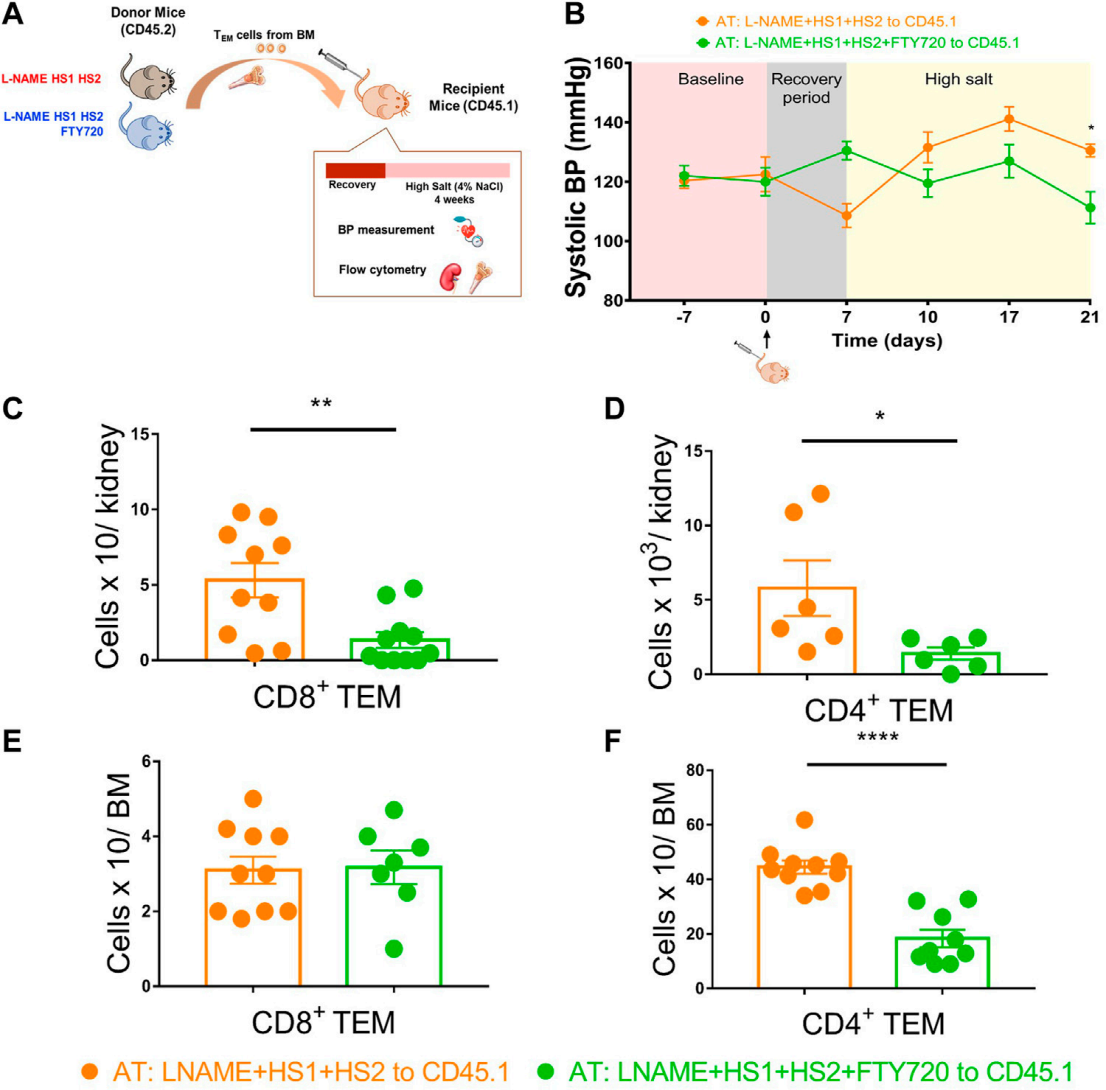
Fingolimod inhibits the recirculation and redistribution of T_EM_ cells into the kidney in response to moderate hypertensive stimulation. **(A)** Experimental paradigm: Summary of the protocol followed to study the effect of fingolimod on the recirculation and redistribution of TEM cells after a moderate hypertensive stimulus. Bone marrow TEM cells from donor CD45.2 WT mice received L-NAME + HS1+HS2±fingolimod were adoptively transferred to recipient CD45.1 mice. Recipient mice were then fed a high salt diet for 3 weeks (N = per group) **(B)** Tail-cuff systolic blood pressure measurements were recorded throughout the whole protocol starting from the baseline (before adoptive transfer) and reaching the end of the 3 weeks of high salt feeding. **(C–F)** After a successful AT, the levels of the transferred CD8^+^ and CD4^+^ TEM cells were measured in the kidney and bone marrow of the recipient mice following high salt feeding. N = 6–11 mice/group. Data are expressed as mean ± SEM. *p*-values for the effect of l-NAME/High Salt as calculated by unpaired *t*-test are shown. *p < 0.05,**p < 0.01, ****p < 0.0001.

Itani et al. showed that repeated antigenic exposure reactivates bone marrow-residing T_EM_ cells. These cells exhibit salt sensitivity when adoptively transferred from CD45.2 mice that had undergone the l-NAME/high salt protocol into naive recipient CD45.1 mice exposed to a high salt diet for 3 weeks. We performed the adoptive transfer to study the effect of S1PR1 pharmacological inhibition, through fingolimod injections, on the reactivation and redistribution of bone marrow-residing T_EM_ cells to the kidney in response to a moderate hypertensive stimulus. Bone marrow T_EM_ cells from CD45.2 mice were harvested, sorted, and then adoptively transferred to CD45.1 naïve recipients. The mice were fed a high salt diet of 4% NaCl for 3 weeks (Figure 5A). Tail cuff BP recordings showed that CD45.1 mice receiving T_EM_ cells from mice exposed to L-NAME + HS1+HS2+fingolimod did not exhibit salt sensitivity and thus were protected from developing hypertension in response to the HS diet (Figure 5B).

In contrast, the group receiving T cells from donors exposed to the L-NAME + HS1 + HS2-fingolimod protocol showed BP elevation more significantly than those who received T cells from mice exposed to L-NAME + HS1+HS2+fingolimod (SBP = 130 mmHg vs. 111.3 mmHg, *p*-value 0.0167) (Figure 5B). We also measured CD4^+^ and CD8^+^ T_EM_ cells accumulation in the kidney of recipient mice; interestingly, CD8^+^ and CD4^+^ T_EM_ cells accumulation was significantly higher in the kidney of the group receiving T cells from donors exposed to L-NAME + HS1+HS2-fingolimod (*p*-value: 0.0041 and 0.0440, respectively) (Figures 5C,D). Surprisingly, when analyzing the T_EM_ cells population of the CD45.1 mice bone marrow, a significant accumulation of CD4^+^ T_EM_ cells was shown in mice that received T cells from donors that were not injected with fingolimod (*p*-value <0.0001) (Figure 5F). Therefore, the CD4^+^ T_EM_ cells did not traffic back to the bone marrow.

## Discussion

In our previous study, we found clear support for the role of T_EM_ cells in elevating blood pressure, triggering renal damage, and increasing susceptibility to moderate hypertension after an initial hypertensive challenge (Itani et al., 2016b). T_EM_ cells sensitive to hypertension may persist in the bone marrow, waiting for recurring periods of hypertension, including emotional stress, catecholamine surges in sleep apnea, or repeated bouts of excessive salt consumption. The results of this study go beyond previous reports, highlighting the role of sphingosine 1-phosphate receptor 1 (S1PR1) in trafficking T_EM_ cells between the bone marrow and kidney upon exposure to hypertensive stimuli. Overall, we showed that pharmacological blockade of the S1PR1 expressed by the T_EM_ cells locks this population in the bone marrow and weakens the severity of hypertension and kidney damage. These results show that T_EM_ cells exhibit salt sensitivity for a prolonged time and S1PR1 blockade by fingolimod inhibits this characteristic.

We adopted the mouse model of l-NAME, followed by high salt feeding, regarding the experimental models. The findings of two long-term studies that looked at the prevalence of cardiovascular events or mortality in patients with salt-sensitive hypertension provided strong evidence that salt-sensitive hypertension is clinically significant (Morimoto et al., 1997; Weinberger et al., 2001). Following up with hypertensive patients revealed that participants with salt-sensitive hypertension experienced fatal or non-fatal cardiovascular-related events twice more than those with salt-resistant hypertension (Morimoto et al., 1997). Therefore, we adopted the model of l-NAME followed by high salt feeding. Although there is a variety of salt-sensitive hypertensive animal models (Elijovich et al., 2016), the model implemented in this study mimics salt-sensitive hypertension among humans. By analyzing this hypertensive model, we found evidence for the associations between changes in dietary sodium intake, accumulation and activation of immune cells in the kidneys and bone marrow, production of IL-17A and IFN–γ, and potential development of salt-sensitive hypertension (Itani et al., 2016b). These findings align with previous results that closely link high blood pressure and aberrant immune response (Drummond et al., 2019).

Molecularly, the S1P signaling pathway was found to regulate cell destiny, vascular tone, endothelial function and integrity, and lymphocyte trafficking; hence any imbalance in its production or signaling has been connected to the development of hypertension. Most of the current evidence supports the predominant role of S1P receptor type 1 (S1PR1), specifically in BP homeostasis, by regulating the vascular tone (Cantalupo et al., 2017) and kidney functioning (Koch et al., 2013; Li and Zhang, 2016). Interestingly, S1PR1 is not only expressed in cardiomyocytes, vascular smooth muscle cells, and endothelial cells of cardiac vessels, but also on lymphocytes. Being expressed on lymphocytes, S1PR1 directs T cells from the low S1P environment in the lymphoid organs towards the blood with a high S1P concentration (Lucaciu et al., 2020b). Thus, it plays a role in T cell trafficking between organs. To determine the role of S1PR1 in hypertension-induced end-organ damage and reactivation and trafficking of T_EM_ cells in response to repeated high salt stimulation, we used the immunosuppressant fingolimod, a functional antagonist for S1PR1. Thus, during the second-high salt feeding period, the mice we divided into two groups, the first group received fingolimod injections every other day while the other group received saline solution.

Results obtained from the previous analysis have implications for assuring that our salt-sensitive hypertensive model is successful. Itani et al. showed that the blood pressure of WT mice increased to 140 mmHg after undergoing the l-NAME/high salt protocol described above (Itani et al., 2016b). An interesting observation in the current study is that fingolimod treatment prevented blood pressure elevation in response to hypertensive stimuli like a high salt diet. As far as we know, many studies assessed fingolimod’s activity in several hypertensive animal models, but their results appeared to be inconsistent. In line with our results, Meissner et al. reported that treatment Ang-II induced hypertensive mice with fingolimod mitigated the development of hypertension after pump insertion (Meissner et al., 2016).

In this study, the mice received fingolimod (1 mg/kg) IP every other day over a period of 2 weeks (Meissner et al., 2016), to block the egress of TEM cells and thus lessen kidney damage. Liang and collegues, reported a decrease in TEM trafficking after denervating the bone marrow as a niche to TEM cells (Xiao et al., 2020). A similar conclusion was reached by Meissner et al., demonstrating that chronic fingolimod administration in pre hypertensive mice triggers peripheral lymphopenia and thus prevents AngII-induced development of HT (Meissner et al., 2016). On the other hand, other studies did not verify fingolimod’s protective effects against hypertension. Cantalupo and colleagues, demonstrated that chronic administration of fingolimod increased the blood pressure of hypertensive mice (Cantalupo et al., 2017). Following different treatment regimens (dose and frequency of treatment administration) and using different hypertensive models may explain these contradicting results (Jozefczuk et al., 2020). Another factor that explains these contradicting results is the time of fingolimod administration in hypertension. To illustrate, at the onset of hypertension, fingolimod was able to protect against blood pressure development, but when given as a “treatment” for hypertension, fingolimod was not protective and even increased blood pressure (Elijovich et al., 2016; Li and Zhang, 2016). Regarding figolimod’s effect on lumphocytes, an INFORMS, multicentre, double-blind, placebo-controlled parallel-group study, showed that primary pregressive MS patients receiving long-term fingolimod treatment (0.5 mg/d over 5 years) decreased the mean absolute lymphocyte count by 70% (Fox et al., 2019). Similarly, other studies reported that short-term and low-dose administartion of fingolimod induced regulatory T-cell response and inhibited effector T responses in mice (Huang et al., 2012). This could be explained by the fact that prolonged administration period of fingolimod, leads to more effective internalization and degradation of S1PR1 molecules on lymphocytes (Sykes et al., 2014). Besides, a pioneeristic work reported that Fingolimod prevents lymphocyte infiltration to the ischemic brain tissue without affecting the infiltration of myeloid cells (Salas-Perdomo et al., 2019). Thereore, we can say that our results might have important clinical implications, yet one concer about the findings of our study was the treatment regime. This can derive future studies to answer an intresting research question on the precise effects of *in vivo* fingolimod treatment on BP in various hypertensive animal models is needed.

Interestingly, S1P has a diverse effect on the vasculature, depending on the dominant receptor subtypes in endothelial cells or smooth muscle cells (Intapad, 2019). S1PR1 and S1PR3 expressed on the endothelial cells are vasodilators, while S1PR2 and S1PR3 extensively expressed on the vascular smooth muscle cells are vasoconstrictors. From another perspective, fingolimod effects on the S1P-S1PR1 axis are species and vessel type-dependent (Intapad, 2019). In epinephrine-preconstricted mesenteric arterioles obtained from rats or mice, S1P causes endothelial nitric oxide synthase-dependent vasorelaxation, rodent cerebral arteries, and mesenteric resistance arteries from elderly rats showed S1P-induced vasoconstriction (Hemmings, 2006; Schuchardt et al., 2011). Additionally, a study reported that the loss of endothelial S1PR1 expression in resistance arteries via fingolimod treatment, abolishes vasorelaxation, increases blood pressure in control mice, and exacerbates hypertension in the Ang-II mouse model (Cantalupo and Di Lorenzo, 2016).

Based on the analysis of several studies, the kidney forms a niche for immune cell infiltration and/or activation in case of hypertension (Johnson et al., 2005; Bravo et al., 2007). Our current findings showed a significant decrease in the infiltration of CD4^+^ and CD8^+^ T_EM_ and T_CM_ cells into the kidney of mice treated with fingolimod compared to the mice group which did not receive fingolimod. In our published study, Itani et al. reported that CD4^+^ and CD8^+^ T_EM_ cells increase in the kidney with an HS diet and re-accumulate significantly with repeated HS stimulation. Miguel et al. reported similar observations in their experiments. Briefly, it was reported that treating hypertensive animals with immunosuppressants prevented the infiltration of T cells into the kidneys and mitigated blood pressure (De Miguel et al., 2011). On the contrary, no blood pressure reduction was reported in control animals upon administering immunosuppressants. Accordingly, pharmacologically blocking S1PR1 by fingolimod, which is considered an immunosuppressant, attenuates the development of hypertension and renal damage in our mouse model. This observation was consistent with that noted among humans (Mattson, 2014). Initial studies reported the presence of T cells in the kidneys of patients with essential hypertension, while hypertensive patients with autoimmune disease who receive immunosuppressants had a better response to hypertensive stimuli (Zhang and Crowley, 2015). The kidneys form a great site for T cells infiltration and activation leading to inflammation in hypertensive models. After observing the decrease in the entry of T_EM_ cells into the kidneys in mice treated with fingolimod, we assessed the renal damage. Our analysis regarding immunostaining of CD3^+^ cells in the kidney assures the infiltration and inflammation of kidneys of mice that did not receive fingolimod, a completely abolished phenomenon in mice treated with fingolimod. In addition to that, since NGAL is a promising biomarker for acute kidney damage, we measured its expression level. NGAL expression significantly decreased in the group treated with fingolimod, suggesting reduced tubular damage and that the fingolimod has a protective effect on the kidney. On another note, Buonafine and colleagues asserted that NGAL has a pathogenic role in cardiac damage besides its role as a biomarker for renal injury. NGAL has a crucial role in aldosterone/MR-dependent hypertension (Buonafine et al., 2018).

Here in, we showed that S1PR1 blockade, by fingolimod treatment, locked the CD8^+^ T_EM_ cells in the bone marrow. Besides, our previous study proved that CD4^+^ and CD8^+^ T_EM_ cells increase in the bone marrow upon l-NAME/high salt exposure. These results suggest that the bone marrow cells contribute to hypertension, as it is a perfect residence for the quiescent T_EM_ (Durlanik et al., 2015). These T_EM_ cells remain quiescent for months and could be reactivated upon repeated treatment antigenic exposure (Itani et al., 2016b). The findings confirm that T_EM_ cells are formed upon exposure to the HS stimulus and reactivated to repeated stimuli. However, the exodus of these cells from the bone marrow was blocked with fingolimod, and the kidney was protected. Our observations agree with Fujii et al., which showed that the number of T cells increases considerably in SPX LT-α−/− mice treated with fingolimod (Fujii et al., 2008). Interestingly, the S1P-S1PR1 axis is known to control T cell egress from bone marrow, and S1PR1 surface abundance on T cells and local S1P concentrations are closely linked (Schwab et al., 2005). In parallel with findings from animal models, an important study by Chongsathidkiet et al., showed that patients with glioblastoma have an abundance of thymocytes in the bone marrow, which is associated with the internalization of sphingosine one phosphate receptor 1 (S1PR1), which plays a key role in thymocyte trafficking. Furthermore, this thymocyte accumulation in the bone marrow was reversed when S1PR1 internalization was blocked, resulting in more tumor-infiltrating leukocytes and enhanced survival (Chongsathidkiet et al., 2018). Therefore, immunotherapies are becoming a prominent area in research, as the immune system could be enhanced to target life-threatening malignancies and repressed to escape inflammatory reactions, by modulating the S1P-S1PR1 axis. Recent studies have emphasized the role of T cell infiltration into the perivascular fat and its role in inducing vascular dysfunction and organ damage (Mikolajczyk et al., 2016). Thus, S1PR1 inhibition by fingolimod was able to protect the vasculature in response to repeated hypertensive stimulation. This study showed a non-significant decrease in infiltrating T cells in the group treated with fingolimod, except for the CD8^+^ T_CM_ cells. These findings could explain why people with chronic renal illness have a higher rate of vascular disease. (Oh et al., 2002).

Our previous study showed that the T_EM_ cells activated in hypertensive mice travel from the kidney to the bone marrow, spleen, and vasculature. Our results in this study suggest that T_EM_ cells traffic back from the bone marrow to the kidney and vasculature, affecting the function of these blood pressure-regulating organs, and that fingolimod treatment attenuates this phenomenon. Thus, the immunosuppressant, fingolimod, was proven to protect against hypertension and renal damage by sequestering inflammatory T cells in the bone marrow. The role of immunological memory in repetitive hypertension has been studied, but the mechanisms of redistribution and reactivation of T cells remain unclear. Thus, to study the immunological memory of T cells against hypertension, we implement CD45.1 WT mice. We aimed to inject T cells expressing S1PR1 in a group of naïve CD45.1 mice and T cells lacking S1PR1 in another group of naïve CD45.1 mice lacking S1PR1 expression and feed them a high salt diet for the first time.

Interestingly, in our adoptive transfer model, recipient mice receiving T cells from donor mice exposed to l-NAME/HS1HS2 protocol with fingolimod had blunted blood pressure and decreased T cells infiltration into the kidney in comparison to those received T cells from donor mice exposed to l-NAME/HS1HS2 only. Thus, these mice were protected from developing hypertension in response to the HS diet because they lack the salt-sensitive T_EM_ cells. Interestingly and in line with our findings, Guzik and colleagues reported that hypertensive response to angiotensin II was restored in RAG-1/mice after adoptive transfer of T cells, but not B cells (Guzik et al., 2007). (Guzik et al., 2007). Our results showed that S1PR1 blockade inhibits T_EM_ cells infiltration into the kidney and bone marrow. S1PR1 contributes to TEM cells’ egress from the bone marrow to the kidney but does not promote the trafficking back of these cells to the bone marrow. Thus, we can conclude that S1PR1 plays a role in the recirculation and redistribution of T_EM_ cells in response to moderate hypertensive stimulation.

## Conclusion

Hypertension is multifaceted and complex and is the primary cause of myocardial infarction, heart failure, and stroke. Despite current medicines, BP management does not achieve an acceptable outcome in all patients, implying the need for new therapeutic targets and a deeper knowledge of underlying mechanisms. The findings of this study show that blockade of S1PR1 through pharmacological means (fingolimod) affects renal vascular function and raises blood pressure. S1P-S1PR1 signaling pathway was shown to form a hypertensive immunological memory by forming long-lived T_EM_ cells that can live for decades in the bone marrow and induce renal illness when reactivated. Interestingly, this process will be shut down in case of fingolimod treatment. Therefore, this study provides a promising approach that aids in reducing the prevalence of salt-sensitive hypertension.

## Data Availability

The original contributions presented in the study are included in the article/Supplementary Material, further inquiries can be directed to the corresponding author.
