# Supplementary material for: Sphingosine 1 phosphate promotes hypertension specific memory T cell trafficking in response to repeated hypertensive challenges
Source: Front Physiol. 2022 Sep 7;13:930487. doi: 10.3389/fphys.2022.930487 (PMC9490048; doi:10.3389/fphys.2022.930487)
Supplement: Supplementary file 1 [file DataSheet1.PDF]

		D'Agostino & Pearson normality test			Shapiro-Wilk normality test		
		K2	*p*-value	Passed normality test?	W	*p*-value	Passed normality test?
L-NAME + HS1 +HS2	Kidney CD8+ TEM cells	3.277	0.1943	Yes	0.8843	0.0551	Yes
	Kidney CD8+ TCM cells	1.747	0.4174	Yes	0.9224	0.2095	Yes
	Kidney CD4+ TEM cells	3.816	0.1483	Yes	0.9051	0.1336	Yes
	Kidney CD4+ TCM cells	2.931	0.2310	Yes	0.8102	0.0090	No
	BM CD8+ TEM cells	3.03	0.2199	Yes	0.8955	0.1161	Yes
	BM CD8+ TCM cells	7.381	0.0250	No	0.8142	0.0075	No
	BM CD4+ TEM cells	3.176	0.2043	Yes	0.8855	0.0223	No
	BM CD4+ TCM cells	3.415	0.1813	Yes	0.8469	0.0201	No
L-NAME + HS1 +HS2+ FTY720	Kidney CD8+ TEM cells	1.323	0.5160	Yes	0.9445	0.5587	Yes
	Kidney CD8+ TCM cells	2.365	0.3066	Yes	0.8811	0.0603	Yes
	Kidney CD4+ TEM cells	3.816	0.1483	Yes	0.9406	0.5885	Yes
	Kidney CD4+ TCM cells	14.23	0.0008	No	0.6801	0.0005	No
	BM CD8+ TEM cells	0.6689	0.7157	Yes	0.8709	0.1258	Yes
	BM CD8+ TCM cells	3.717	0.1559	Yes	0.8574	0.1131	Yes
	BM CD4+ TEM cells	8.017	0.0182	No	0.8311	0.0124	No
	BM CD4+ TCM cells	1.016	0.6016	Yes	0.9088	0.3074	Yes

Supplementary Table 1: Results of the D'Agostino & Pearson and Shapiro-Wilk normality tests done on data obtained from WT mice following the L-NAME+HS1+HS2±FTY720. All data are normally distributed except for kidney CD4+ TCM cells, BM CD8+ TCM, and CD4+ TEM cells (highlighted in yellow).

		D'Agostino & Pearson normality test			Shapiro-Wilk normality test		
		K2	*p*-value	Passed normality test?	W	*p*-value	Passed normality test?
AT: L-NAME+ HS1+HS2 to CD45.1	Kidney CD8+ TEM cells	2.925	0.2317	Yes	0.9027	0.2348	Yes
	Kidney CD8+ TCM cells		N too small		0.7854	0.0293	No
	Kidney CD4+ TEM cells		N too small		0.8302	0.1079	Yes
	Kidney CD4+ TCM cells		N too small		0.9019	0.3429	Yes
	BM CD8+ TEM cells	1.799	0.4068	Yes	0.8878	0.1601	Yes
	BM CD8+ TCM cells	2.912	0.2332	Yes	0.8972	0.1707	Yes
	BM CD4+ TEM cells	4.816	0.0900	Yes	0.9089	0.2737	Yes
	BM CD4+ TCM cells		N too small		0.9138	0.4229	Yes
AT: L-NAME+ HS1+HS2+ FTY720 to CD45.1	Kidney CD8+ TEM cells	4.122	0.1273	Yes	0.7799	0.0051	No
	Kidney CD8+ TCM cells	2.174	0.3372	Yes	0.9075	0.2985	Yes
	Kidney CD4+ TEM cells		N too small		0.8979	0.3618	Yes
	Kidney CD4+ TCM cells	0.4218	0.8099	Yes	0.9679	0.8807	Yes
	BM CD8+ TEM cells		N too small		0.9599	0.8178	Yes
	BM CD8+ TCM cells	1.714	0.4245	Yes	0.9107	0.2859	Yes
	BM CD4+ TEM cells	2.081	0.3533Yes	Yes	0.8469	0.0689	Yes
	BM CD4+ TCM cells		N too small		0.9816	0.9591	Yes

Supplementary Table 2: Results of the D'Agostino & Pearson and Shapiro-Wilk normality tests done on data obtained from CD45.1 recipient mice. All data are normally distributed except for kidney CD8+ TCM cells (highlighted in yellow).

		Shapiro-Wilk normality test		
		W	*p*-value	Passed normality test?
L-NAME+ HS1+HS2	BM CD8+ TEM IL-17	0.9382	0.6532	Yes
	BM CD8+ TEM IFNγ	0.8181	0.1130	Yes
	BM CD4+ TEM IL-17	0.9935	0.9903	Yes
	BM CD4+ TEM IFNγ	0.8817	0.3169	Yes
L-NAME+ HS1+HS2+ FTY720	BM CD8+ TEM IL-17	0.9494	0.7326	Yes
	BM CD8+ TEM IFNγ	0.8602	0.2291	Yes
	BM CD4+ TEM IL-17	0.8747	0.2859	Yes
	BM CD4+ TEM IFNγ	0.9482	0.7246	Yes

Supplementary Table 3: Results of the D'Agostino & Pearson and Shapiro-Wilk normality tests done on data obtained from Intracellular staining of IL-17 and IFN-γ secreted by CD4+ and CD8+ effector memory cells isolated from mice that received L-NAME+HS1+HS2±FTY720. All data are normally distributed.
